# Surgical Diseases Management during COVID-19 Crisis at a Tertiary Care Hospital of India: Our Institutional Strategy

**DOI:** 10.1055/s-0041-1740452

**Published:** 2021-12-28

**Authors:** Sudhir Kumar Singh, Amit Gupta, Harindra Sandhu, Rishit Mani, Jyoti Sharma, Praveen Kumar, Deepak Rajput, Navin Kumar, Farhanul Huda, Som Prakas Basu, Bina Ravi, Ravi Kant

**Affiliations:** 1 Department of Surgery, All India Institute of Medical Sciences, Rishikesh, Uttarakhand, India

**Keywords:** COVID-19, pandemic, elective, emergency, surgery

## Abstract

**Introduction** In response to the national coronavirus disease 2019 (COVID-19) pandemic, all hospitals and medical institutes gave priority to COVID-19 screening and to the management of patients who required hospitalization for COVID-19 infection. Surgical departments postponed all elective operative procedures and provided only essential surgical care to patients who presented with acute surgical conditions or suspected malignancy. Ample literature has emerged during this pandemic regarding the guidelines for safe surgical care. We report our experience during the lockdown period including the surgical procedures performed, the perioperative care provided, and the specific precautions implemented in response to the COVID-19 crisis.

**Materials and Methods** We extracted patient clinical data from the medical records of all surgical patients admitted to our tertiary care hospital between the March 24th, 2020 and May 31st, 2020. Data collected included: patient demographics, surgical diagnoses, surgical procedures, nonoperative management, and patient outcomes.

**Results** Seventy-seven patients were included in this report: 23 patients were managed medically, 28 patients underwent a radiologic intervention, and 23 patients required an operative procedure. In total eight of the 77 patients died due to ongoing sepsis, multiorgan failure, or advanced malignancy.

**Conclusion** During the COVID-19 lockdown period, our surgical team performed many lifesaving surgical procedures and appropriately selected cancer operations. We implemented and standardized essential perioperative measures to reduce the spread of COVID-19 infection. When the lockdown measures were phased out a large number of patients remained in need of delayed elective and semi-elective operative treatment. Hospitals, medical institutes, and surgical leadership must adjust their priorities, foster stewardship of limited surgical care resources, and rapidly implement effective strategies to assure perioperative safety for both patients and operating room staff during periods of crisis.

After the official documentation of cases of pneumonia of unknown etiology on December 31 ^st^ , 2019 by Wuhan Municipal Health Commission, the offending virus identified was named as severe acute respiratory syndrome coronavirus 2 (SARS-CoV-2). 1 The World Health Organization (WHO) named this novel disease as coronavirus disease 2019 (COVID-19) on February 11, 2020 and declared it as a pandemic on March 11, 2020, after the rapid spread of this disease globally. 2 Now more than 200 countries have been affected with its outbreak and it has become a matter of public health concern as it is a highly contagious disease which spreads via air droplet during speaking, sneezing, and coughing. 3

On January 30, 2020 India became a part of this global carnage with the detection of its first COVID case. 4 To curtail the infection and spread of disease, Government of India (GOI) declared a nationwide lockdown of 21 days from March 24 ^th^ , 2020. Since then every patient coming to hospital was being screened for COVID-19 before admission as per the institute's policy. On April 14th, GOI announced the extension of the nationwide lockdown till May 3rd owing to the rising number of cases in the country. It was further extended till May 17th (Lockdown 3.0) and subsequently till May 30th (Lockdown 4.0) while giving successive relaxation. 5 6 As the priority of health care has changed to cater only emergency and semi-emergency conditions, it has also affected the care provided by surgical disciplines. We report our experience regarding the effect of COVID lockdown on the spectrum of surgical diseases encountered, their management, clinical outcomes along with an emphasis on the precautions taken to minimize the spread of infection to health care workers (HCWs).

## Material, Method, and Statistical Analysis

Clinical data of all patients admitted in Department of Surgery from March 24th to May 31st, was extracted from the medical records. Demographic profile of patients, their diagnosis, management, and outcomes were analyzed by SPSS v.26.0. Length of the hospital stay, morbidity (according to Modified Clavien-Dindo [CD] Classification), and mortality were recorded. Descriptive analysis was done by calculating the arithmetic mean, mode, and percentile.

## Results

A total of 77 patients were admitted in the surgery ward out of which 54 were males and 23 were females. The average age of the patients was 44.37 (SD) years with two patients of more than 74 years of age. Total number of patients with associated comorbidities were 29 (37.6%). ( Table 1 )

**Table 1:** Sociodemographic profile of surgical patients

	Number of patients (%)
Age groups (years)	
18–59	56 (72.7%)
60–74	19 (24.6%)
> 74	2 (2.5%)
Gender	
Male	54 (70.1%)
Female	23 (29.8%)
Co-morbidities	
Coronary artery disease (CAD)	7 (9.1%)
Hypertension (HTN)	7 (9.1%)
Chronic obstructive pulmonary disease (COPD)	3 (3.8%)
Diabetes mellitus (DM)	8 (10.8%)
DM with HTN	4 (5.2%)
GI malignancies	
Esophagus	1 (1.3%)
Stomach	1 (1.3%)
Duodenum	1 (1.3%)
Colon	1 (1.3%)
Hepatopancreatico-biliary malignancies	
Gall bladder	5 (6.5%)
Cholangiocarcinoma	3 (3.8%)
Periampullary	1 (1.3%)
Head of pancreas	2 (2.5%)
Breast and endocrine	
Breast	4 (5.2%)
Benign diseases	
Hollow viscus perforation with peritonitis	14 (18.1%)
Sub-acute intestinal obstruction	5 (6.5%)
Koch's abdomen	1 (1.3%)
Enterocutaneous fistula	1 (1.3%)
Mesenteric Ischemia	1 (1.3%)
Acute pancreatitis	3 (3.8%)
Liver abscess	8 (10.3%)
Acute cholecystitis	2 (2.5%)
Cholelithiasis with choledocholithiasis	4 (5.2%)
Biliary fistula	1 (1.3%)
Cholangitic abscess	1 (1.3%)
Acute appendicitis	1 (1.3%)
Foreign body ingestion	1 (1.3%)
Incisional hernia	2 (2.5%)
NSTI	4 (5.2%)
Surgical site infection (SSI)	2 (2.5%)
Peripheral vascular disease	2 (2.5%)
Psoas abscess	1 (1.3%)

**Fig. 1:**
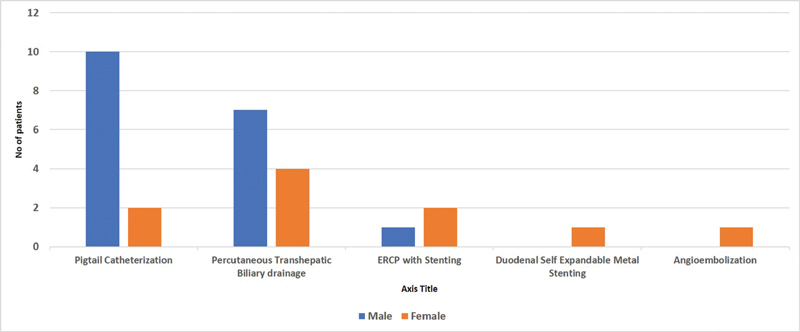
Radiological interventions done in surgical patients.

Out of 77 patients, 58 (75.3%) presented with benign diseases, majority being hollow viscus perforation (14 patients, 24.1%) and liver abscess (8 patients, 13.7%). The number of patients presenting with malignancy were 19 (24.6%), out of which hepatopancreato-biliary malignancy formed a major part, i.e., 11 patients (57.8%). Radiological intervention was required in 28 patients that commonly included ultrasound-guided pigtail catheterization and percutaneous transhepatic biliary drainage (PTBD). Surgical conditions requiring pigtail catheterization included liver abscess, cholangitic abscess, deep space surgical site infection, and sealed off perforation. PTBD was mainly done as a palliative procedure for obstructive jaundice due to hepatobiliary and duodenal malignancies and in one case of choledocholithiasis-induced severe cholangitis. Endoscopic retrograde cholangiopancreaticography and stenting was done for biliary fistula, as a palliative procedure in metastatic pancreatic cancer and for the removal of retained T-tube fragment. Endoscopic-guided self-expandable metallic stenting was done for a patient with gastric outlet obstruction due to local recurrence of carcinoma gall bladder infiltrating the first part of duodenum and angio-embolization was done in a patient with post-pancreatitis gastroduodenal artery aneurysm ( Fig. 1 ). Twenty three out of 77 patients were operated for emergency and semi-emergency conditions, out of which two cases were of breast malignancy. One patient with gastric outlet obstruction due to metastatic duodenal adenocarcinoma was planned for palliative surgery, but expired during initial resuscitation due to antecedent dyselectrolemia and dehydration. All patients were classified under ASA grading on pre-anesthetic check-up ( Table 2 ). Patients requiring surgical intervention were taken up for surgery as per institutional operative protocols. Out of 14 patients presenting with peritonitis due to hollow viscus perforation, exploratory laparotomy was done in 13 patients while one patient expired during initial resuscitation. Two patients presented with acute intestinal obstruction due to postoperative adhesions and obstructed incisional hernia which was managed by surgical intervention. Two out of four patients with obstructive jaundice due to CBD stones required surgical intervention whereas one patient with severe cholangitis was managed by PTBD and the other was discharged on request after resolution of symptoms. One patient with wet gangrene of lower limb underwent below knee amputation.

**Table 2:** Surgical procedures and outcome of patients

	Number of patients
ASA grade	
I	6
II	14
III	3
IV	0
V	0
Surgical procedures	
Modified GPR	8
Modified GPR with RD & FJ	2
Modified GPR with RD, gastrostomy & FJ	1
Exploratory laparotomy with double-barrel ileostomy	2
Exploratory laparotomy with adhesiolysis	1
Open cholecystectomy, CBD exploration with T-tube	2
Debridement and incision and drainage	3
Below knee amputation	1
Primary repair of hernia defect	1
Left MRM with LD flap	2
Modified Clavien-Dindo classification (postoperative outcome)	
I	8
II	6
III	1
IV	5
V	3

Abbreviations: FJ, feeding jejunostomy; GPR, Graham's patch repair; LD flap, latissimus dorsi flap; MRM, modified radical mastectomy; RD, retrograde duodenostomy.

Postoperative outcome of patients was categorized according to Modified CD classification. Out of 23 operated patients, one patient required USG-guided pigtail catheterization (CD grade III) and five patients needed intensive care unit (ICU) care (CD grade IV). Three cases operated for ileal perforation, prepyloric peptic perforation, and anterior abdominal wall NSTI succumbed to their illness due to septicemia and multiorgan failure.

Two out of 77 patients were confirmed as COVID positive, out of which one was diagnosed with severe acute pancreatitis with septic shock and multiorgan dysfunction while the other was a case of acute chronic pancreatitis. The former succumbed to her illness while the latter was discharged after 30 days with a negative COVID report.

The average length of hospital stay during this period was 10.7 days. This was due to the long waiting period for COVID-19 test result and mandatory quarantine. Eight mortalities were noted in this duration which included two patients of carcinoma breast and one patient with metastatic cholangiocarcinoma, both of which succumbed to the terminal stage of the disease while five patients expired due to septicemia and multiorgan failure. These five included three postoperative patients, one patient with severe acute pancreatitis with COVID positive status and one with cholangitic abscess.

## Discussion

During national lockdown, the Ministry of Health and Family Welfare issued guidelines for hospitals to ensure resource preservation and safety of HCWs on account of rapid spread of COVID infection. 7 Resource preservation was necessary to handle the worsening pandemic situation so that its allocation could be done to frontline health workers in emergency department and ICU, if required. Various principles were adopted by general surgeons in this pandemic phase:

1. Postponement of elective surgeries except the diseases which were time-sensitive like malignancy or limb salvage surgery.
2. Only emergency cases to be catered and prompt management to be done by the designated surgical team, that too with adequate personal protective equipment (PPE).
3. Mandatory COVID testing for all cases before surgery by reverse transcription polymerase chain reaction (RT-PCR) test.
4. Avoiding aerosol generating procedures like laparoscopy, endoscopy, and robotic surgeries. When mandatory, full PPE including N95 respirators must be worn by surgeons and operating room (OR)/endoscopy staff.
5. Minimum possible staff must be kept to reduce the consumption of PPE and exposure to high-risk procedures.
6. Risk stratification of patients is important to avoid over-depletion of PPE supplies. 8 9 10

Our institute was focused on catering the emergency and semi-emergency cases during the lockdown period. All outpatient services and elective surgeries were suspended immediately. Only patients needing COVID testing and acute care were allowed to enter the hospital premises. High-risk individuals were sampled via nasopharyngeal and oropharyngeal swab and were advised home-based quarantine for 14 days. 11 Patients requiring acute medical or surgical care and patients with malignancy were scrutinized by the institute's screening team, along with their attendants, for symptoms related to COVID-19. If the COVID screening test (based on symptoms and associated risks of COVID-19) was negative, the patients were allowed to consult the non-COVID emergency department for the evaluation and further management. All COVID suspects were admitted in the COVID emergency ward where initial treatment was given and a nasopharyngeal swab was taken for RT-PCR test. 12 13 The waiting period for COVID test result was approximately 24 hours. If the test result was negative for COVID, patients were shifted to general ward of respective departments for further evaluation and management while COVID positive patients were kept in COVID ward or ICU according to the need and were managed accordingly. Retesting of COVID positive patients was done after 13 days of the first sample during the same hospital admission. Follow-up of patients was done telephonically via telemedicine OPD and the follow-ups of patients with time sensitive diseases requiring surgery were planned accordingly. However, many patients faced difficulty in traveling due to lockdown constraints and thus, were lost to follow-up.

Nineteen out of 77 patients presenting with acute surgical condition needed emergency or urgent surgery (i.e., requiring surgery within 12 hours) and were operated in COVID OR as their test results were awaited. These patients were shifted to general ward or ICU after negative COVID test result. Four cases, two patients with carcinoma breast and two with choledocholithiasis, were operated electively in routine OR with full precautions as per institution protocol

### Operative Protocol for Suspected or Confirmed COVID-19 Cases

**Fig. 2:**
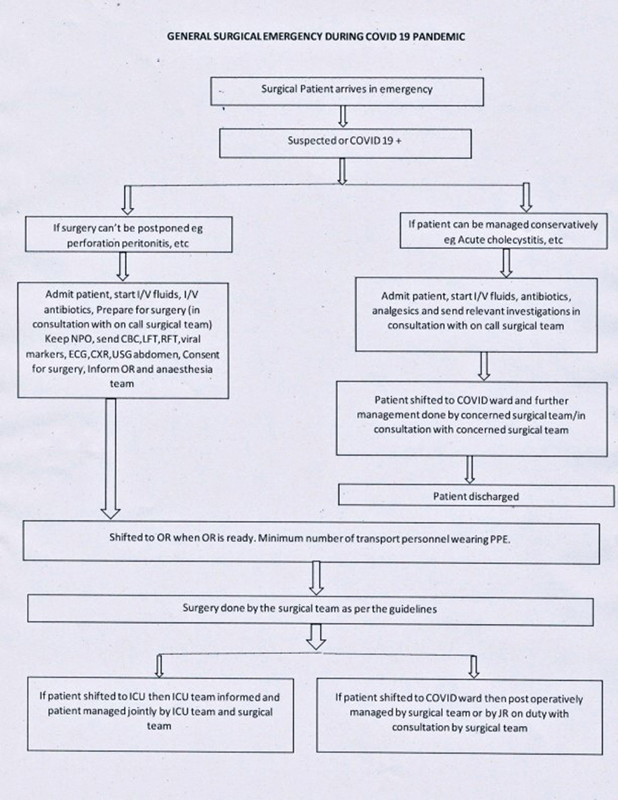
Algorithm used to define the management of patients requiring surgical intervention.

A multidisciplinary team comprising of surgeons, anesthetists, physicians, critical care specialists, and nursing supervisors formulated an Institutional operative protocol which outlined ( Fig. 2 ) the management and preventive measures to be taken during the perioperative period in suspected or confirmed COVID-19 patients requiring surgery. 14 15 16

The following protocol was followed for operative patients

### Preoperative Phase

After written and informed consent explaining the risk of contracting COVID infection during the perioperative period, the patients were shifted to COVID ward or ICU for initial resuscitation. The patients were transported via a predetermined, shortest route with dedicated hospital staff after ensuring no hindrance during transportation. The patients were required to wear a disposable head cap, three-layered mask and shoe covers during transportation which was initiated only after confirming the preparedness of surgical team.

### Intraoperative Phase

**Fig. 3:**
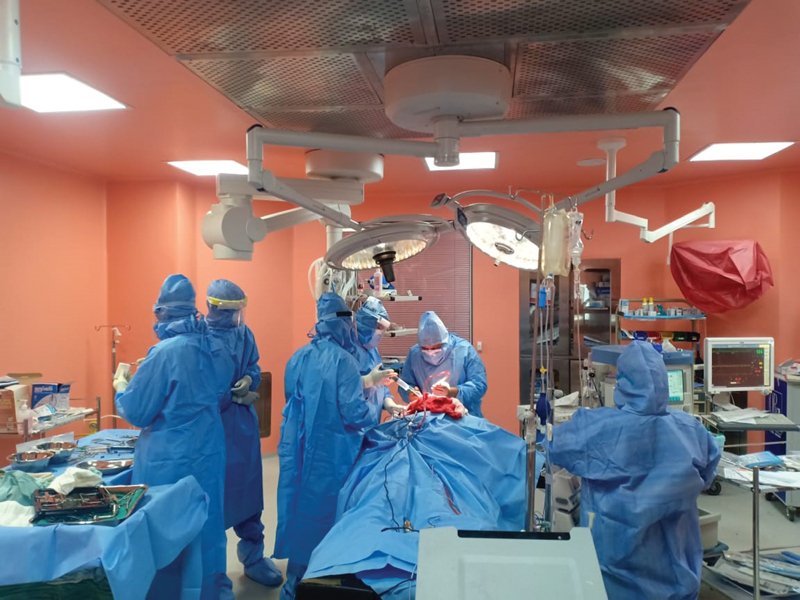
Surgical team in personal protection equipment in operating room.

A dedicated modular OR was designated for COVID patients with modifications to maintain negative air pressure, thus minimizing the risk of transmission of COVID. The operating team along with the anesthetists were kept to a minimum and unnecessary movement and traffic were restricted ( Fig. 3 ). Entry and exit information of every member of operating team was maintained for contact tracing, if needed. Only essential equipment were allowed inside the OR. Use of energy devices was kept to a minimum. Appropriate smoke and gas evacuation systems were utilized for filtration of smoke and aerosols with possible viral particles. Induction of anesthesia was performed with minimum personnel using disposable equipment as per anesthesia guidelines, taking care that the production of aerosols was kept to a minimum. The operating team was advised to keep out of the OR during induction and to enter 15 minutes after intubation. Caution was taken to minimize spillage and contamination by blood or body fluids during operation. The surgical specimen was carefully packed and transported to the pathology department.

### Postoperative Phase

Appropriate measures were taken to decrease the chances of aerosol formation during extubation and the patient was shifted to the recovery room only after full recovery from anesthesia. Transportation of patient out of the OR complex was done in a similar manner as the preoperative transportation. Used PPE was removed in the doffing area as per instructions for PPE removal before going to the clean area. It was mandatory to change the scrubs after each procedure and take a shower, whenever possible. The OR was kept vacant for 60 minutes to allow proper air exchange followed by cleaning and fumigation as per the institutional protocol. Disinfection of OR and the surrounding areas along with patient transit areas was done by dedicated staff in full PPE. For waste disposal, separate containers were placed inside the OR and in the doffing area. All contaminated material and PPEs were sent immediately to a collection point for proper disposal as per protocol.

As directed by the GOI, all government and private hospitals were allowed to provide health care during lockdown after ensuring proper safety measures. But several patients suffered delay in treatment due to lockdown constraints, unawareness about provision of essential health services by hospitals, the perceived fear of contracting COVID-19, or reluctance of private hospitals to provide care. This resulted in increased morbidity, chance of mortality, and longer length of hospital stay.

### Special Consideration for Minimal Invasive Surgeries

At most surgical centers, management during this pandemic comprised of open surgical approach with total abandonment of minimally invasive surgeries (MIS) due to higher presumed risk of transmission of the virus. But there has been a constant debate among the surgeons primarily due to the lack of a definitive evidence regarding the spread of the virus. 17 18

Opponents of MIS have been arguing on the basis of the assumption that aerosol formation will be relatively more due to pneumoperitoneum and the leaks through port site during transference of the instruments, which cannot be completely eliminated. Proponents, on the other hand, are defending the rationality of minimal access surgery during this pandemic with the argument that it has a potential for filtration of confined aerosolized particles in the peritoneal cavity which is difficult in open surgery. So, in spite of less aerosol generation expected in open surgery, the chances of exposure are more due to free dispersion. 19 20 21 22

Various surgical organizations like SAGES and ACS and teaching institutions have released amended recommendations and guidelines for surgical care during this pandemic. 23 24 Our institution is currently following the mentioned guidelines:

1. Avoidance of hand-assisted laparoscopic surgery.
2. Pneumoperitoneum should be created via closed technique and intra-abdominal pressure should be maintained between 10 and 12 mm Hg.
3. Insufflation of CO _2_ should be done at a minimum flow rate.
4. The incision for ports should be just adequate for the passage of trocar to prevent leaks around the ports.
5. Minimum number and size of ports should be used. Proper air seal must be ensured.
6. Pneumoperitoneum should be evacuated via a smoke evacuation and filtration system.
7. Specimen retrieval should be done using an endo bag. The bag should be kept below the valve of the retrieval trocar and should finally be extracted after complete desufflation.
8. The insufflator port should be closed prior to the removal of tubing from the port.
9. Port site closure after complete desufflation of pneumoperitoneum.

### Post Lockdown Period

Gradual unlocking of the country saw a rise in the number of COVID positive patients and related deaths. 25 The main concern was how to restart general outpatient services and elective surgeries without the risk of contracting or spreading infection to the HCWs. The national lockdown resulted in postponement of elective surgeries which were planned to be rescheduled after the resumption of normal OR function. With the resumption of elective OR, provisions were made to tackle the backlog of the elective cases. Telemedicine consultation was started for follow-up of the patients and rescheduling of elective surgeries. The deployment of residents and staff to COVID areas and conversion of surgical facilities to COVID-specific wards became a hindrance to resume a full-fledged surgical care. Physical distancing became the utmost priority to prevent the spread of COVID infection among the HCWs and patients even after resumption of hospital function. Minimal invasive surgeries were performed only in select cases due to the possible risk of transmission of COVID-19 via aerosol production, although a concrete evidence was lacking. 26 27

### Resurgence of “The Second Wave”

The end of the year 2020 saw a gradual decline in the number of COVID-19 cases and resumption of routine surgeries. But the end of March 2021 saw an exponential rise in the number of COVID-19 cases which was due to more transmissible mutants of SARS-CoV-2 virus. Despite the initiation of the COVID vaccination drive, this mutant variant hit like a tsunami in India and had a variable presentation when compared with the SARS-CoV-2 virus. It was due to the fact that this mutant virus showed a widespread disregard to the “COVID appropriate behavior” and was majorly responsible for the second hit. The second wave noted more cases with breathlessness, newer gastrointestinal symptoms and affected the younger and pediatric population.

We implemented the same institutional protocol that was used during the first wave which included emergency surgical care only, while deferring the elective and outpatient cases. The response to the second wave was better than the first wave as the health care system was well prepared this time. The well-trained and experienced HCWS managed the COVID as well as the surgical cases in a better manner, without fear and stress. Preparedness after the first wave like exclusive vaccination drive for HCWs, stocking of the resources like PPE and medications related to COVID care, improving RT-PCR testing facilities helped enormously in tackling the second wave. Minimal invasive surgeries were continued during the second wave as per institutional protocol.

## Conclusion

COVID-19 has taken a huge toll on the country's economy as well as the health care system. With the implementation of nationwide lockdown, the hospitals were focused primarily on dealing with the patients affected with COVID while suspending the outpatient services. The surgical department at our institute was involved in performing lifesaving procedures or selected cancer surgeries while postponing the elective surgeries. This was done for the resource preservation for COVID patients and to decrease the risk of exposure and transmission of COVID-19 to the HCWs. While it was a decent strategy to control the spread of COVID-19, it also led to delay in the management of patients not fulfilling the criteria for emergent care and led to upstaging or increased severity of the disease. Thus the lockdown served as a double-edged sword in the face of health care system. The phased unlocking revealed an enormous backlog of patients requiring management which resulted in an added stress to the already strained health care system. The major concern in post lockdown period was to effectively manage the backlog along with the judicious use of heath care resources to maximize the benefit and reduce the health care burden. Minimal invasive approaches were continued with additional safety precautions, suitable equipment, and expertise. With the anticipation of the “third wave,” preparedness remains the most essential aspect in dealing with a pandemic as one may expect that the worse is yet to come.
